# Supplementary material for: Charge carrier mapping for Z-scheme photocatalytic water-splitting sheet via categorization of microscopic time-resolved image sequences
Source: Nat Commun. 2021 Jun 17;12:3716. doi: 10.1038/s41467-021-24061-4 (PMC8211828; doi:10.1038/s41467-021-24061-4)
Supplement: Supplementary file 1 — Supplementary information [file 41467_2021_24061_MOESM1_ESM.pdf]

Supplementary Information

Charge Carrier Mapping for Z-scheme Photocatalytic Water-Splitting Sheet via Categorization of Microscopic Time-resolved Image Sequences

Makoto Ebihara[1] Takeshi Ikeda,[2,3] Sayuri Okunaka,[2,3] Hiromasa Tokudome,[2,3] Kazunari Domen,[4,5] and Kenji Katayama[1*]

[1] Department of Applied Chemistry, Chuo University, Tokyo 112-8551, Japan

2 Research Institute, TOTO Ltd., Kanagawa 253-8577, Japan

3 Japan Technological Research Association of Artificial Photosynthetic Chemical Process (ARPChem), Tokyo 101-0032, Japan

4 Research Initiative for Supra-Materials, Interdisciplinary Cluster for Cutting Edge Research, Shinshu University, Nagano 380-8553, Japan

5 Office of University Professors, The University of Tokyo, Tokyo 113-8656, Japan

*Corresponding author:

K. Katayama, Phone: +81-3-3817-1913, E-mail: kkata@kc.chuo-u.ac.jp

The role of ITO mediator

It has been reported that the ITO mediator, corresponding to the charge transfer mediator, proceeds the recombination between the electrons in oxygen evolution photocatalyst (OEP) and the holes in hydrogen evolution photocatalyst (HEP), and the efficiency of oxygen and hydrogen evolution rate was drastically improved in this system by inserting this in-between. Therefore, we compared two Z-scheme systems with ITO (STOR/ITO/BVOM) and without ITO (STOR/BVOM) to confirm the effect of the ITO mediator. Supplementary Figure S1 compares the responses of the refractive index change for STOR/ITO/BVOM and STOR/BVOM in ACN. From these responses, we could observe a delayed rising component of around 10 µs in STOR/BVOM, and this component was similar to the Rh^{4+} formation for the STOR only in ACN (refer to Fig.2(c)).

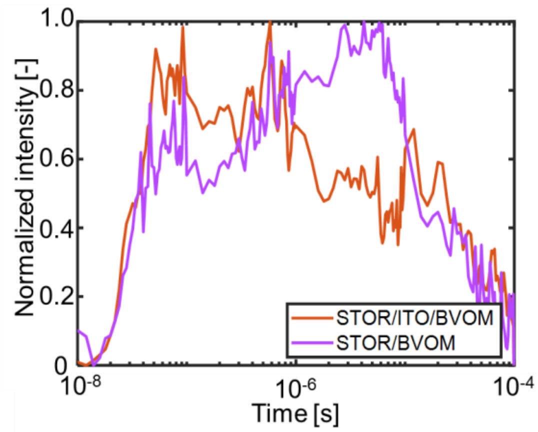

Supplementary Figure 1. **Effect of charge transfer mediator.** The comparison of the refractive index change responses between the Z-scheme sample systems with a charge mediator, indium tin oxide (ITO) for $SrTiO_3$:Rh/ITO/$BiVO_4$:Mo (STOR/ITO/BVOM) (red), and without ITO (STOR/BVOM) (purple) in acetonitrile (ACN) until 100 µs, obtained by the image sequences obtained by the patterned-illumination time-resolved phase microscope (PI-PM).

The difference between these two systems was only the insertion of the ITO mediator. Hence, a possible factor for this difference originated from the charge transfer efficiency of electrons in BVOM and the holes in STOR because the recombination between the electrons in OEP and the holes in HEP was retarded, and they remained in each material without the ITO mediator. Moreover, judging from the similarity in the responses between the STOR/BVOM and the STOR only, the rising component observed in STOR/BVOM must be related to the hole accumulation to the Rh^{3+} state (Rh^{4+} formation) in STOR. This component can possibly be scavenged by a hole scavenger (MeOH) if this assumption is correct. Supplementary Figure 2 shows the refractive index change response for the

STOR/BVOM system in ACN (inert solvent) and MeOH. The result clearly showed that the slow rising component until 10 μs in STOR/BVOM in ACN disappeared in MeOH, and instead, the faster-rising response until 1 μs showed up, which was similar to that of STOR/ITO/BVOM in ACN. It is supposed that MeOH worked to reduce the Rh^{4+} state, instead that the electrons in BVOM were used to reduce it with an ITO mediator. These facts verified that the ITO mediator worked to prevent the increase in the Rh^{4+} states in STOR and proceed the water-splitting reaction efficiently.

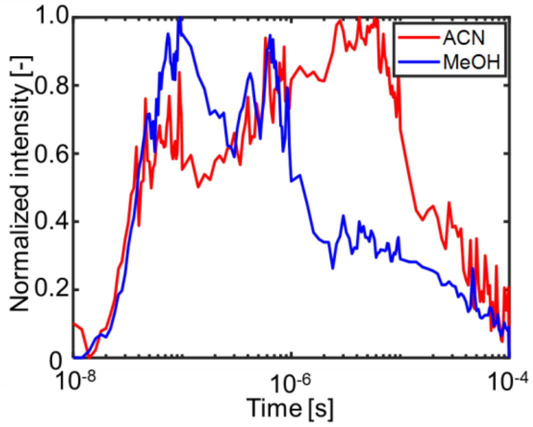

Supplementary Figure 2. **Solvent effect in the absence of charge transfer mediator.** The refractive index change responses for the Z-scheme system, $SrTiO_3$:Rh/$BiVO_4$:Mo (STOR/BVOM) without the indium tin oxide (ITO) mediator in acetonitrile (ACN) (red) and methanol (MeOH) (blue) until 100 μs, obtained by the image sequences of the patterned-illumination time-resolved phase microscope (PI-PM).

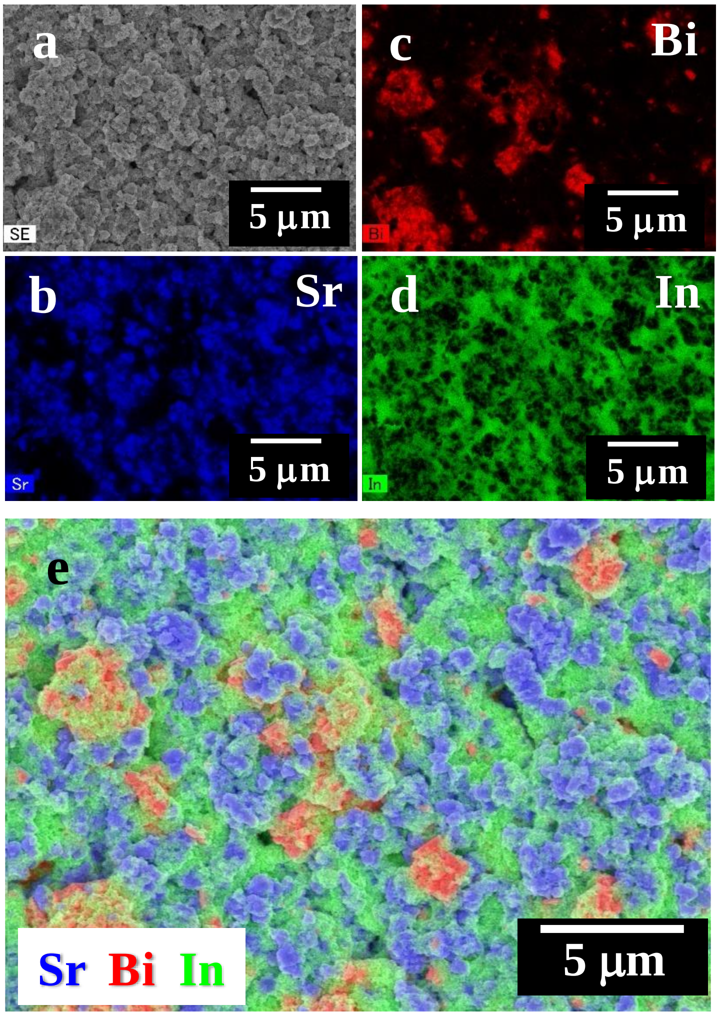

Supplementary Figure 3. **Microscopic images and element analyses.** **a** A scanning electron microscope (SEM) image of a $SrTiO_3$:Rh/ITO/$BiVO_4$:Mo (STOR/ITO/BVOM) photocatalyst sheet and the mapping images of energy dispersive X-ray spectroscopy (EDS) exhibiting **b** Sr, **c** Bi, and **d** In distributions, and **e** a superimposition of the distributions.

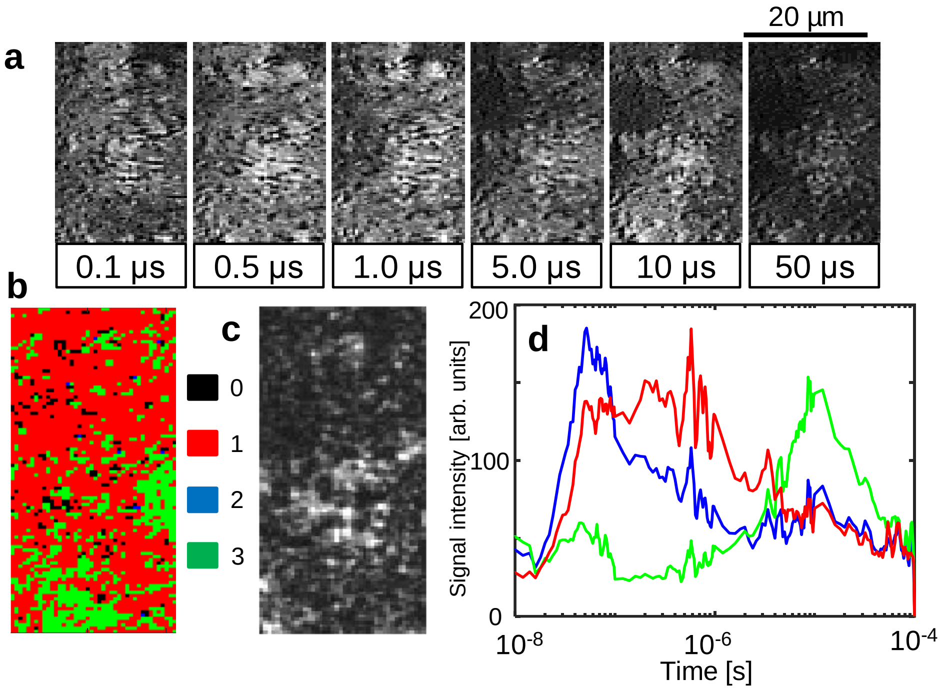

Supplementary Figure 4. **Image sequence and clustering analysis.** (a) An image sequence of the refractive index response for $SrTiO_3$:Rh/ITO/$BiVO_4$:Mo (STOR/ITO/BVOM) in acetonitrile (ACN) in a square region (20×50 µm) corresponding to No.2 in Fig.1(c) on the order from nanoseconds to microseconds. The scale bar corresponds to 20 µm. (b) The categorized mapping of the charge carrier responses of (a). An outlier positioned far from all categories was colored in black (#0). (c) A microscopic image in the same area as (a). (d) The averaged responses for each category in (b) are shown. (red: category 1, blue: category 2, green: category 3)

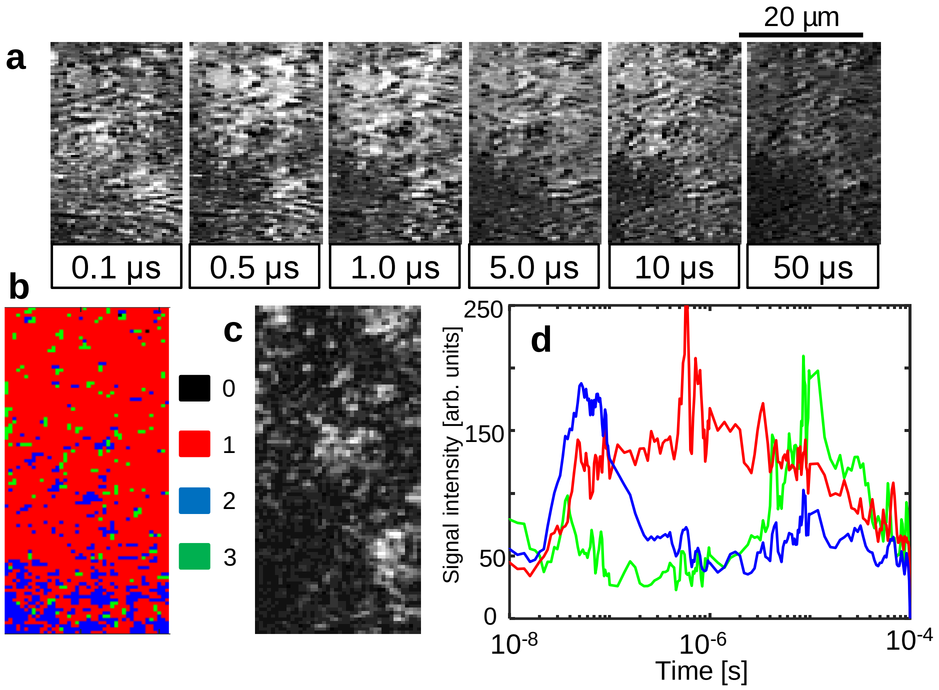

Supplementary Figure 5. **Image sequence and clustering analysis.** (a) An image sequence of the refractive index response for $SrTiO_3$:Rh/ITO/$BiVO_4$:Mo (STOR/ITO/BVOM) in acetonitrile (ACN) in a square region (20×50 μm) corresponding to the region No.3 in Fig.1(c) on the order from nanoseconds to microseconds. The scale bar corresponds to 20 μm. (b) The categorized mapping of the charge carrier responses of (a). An outlier positioned far from all categories was colored in black (#0). (c) A microscopic image in the same area as (a). (d) The averaged responses for each category in (b) are shown. (red: category 1, blue: category 2, green: category 3)

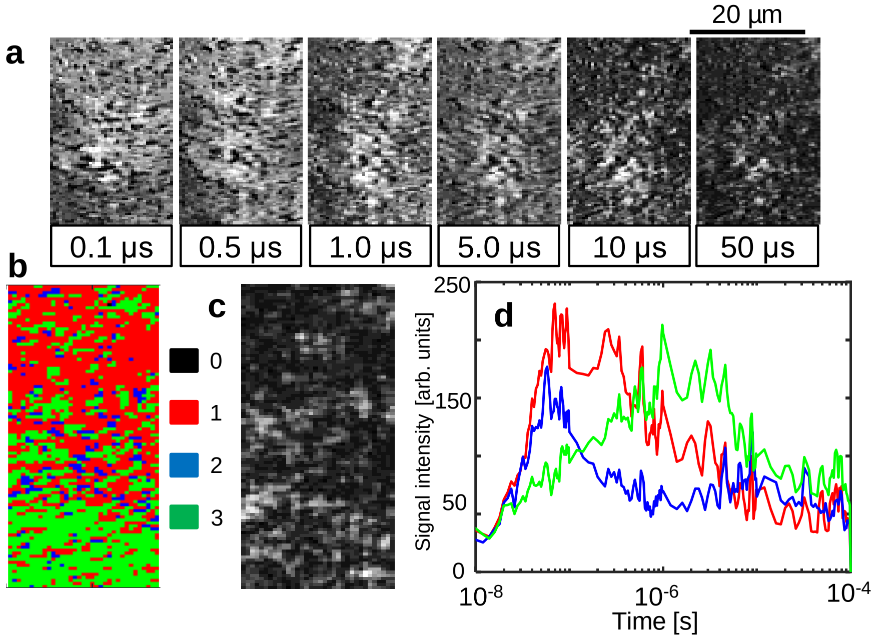

Supplementary Figure 6. **Image sequence and clustering analysis.** (a) An image sequence of the refractive index response for $SrTiO_3$:Rh/ITO/$BiVO_4$:Mo (STOR/ITO/BVOM) in acetonitrile (ACN) in a square region (18×50 µm) corresponding to the region No.4 in Fig.1(c) on the order from nanoseconds to microseconds. The scale bar corresponds to 20 µm. (b) The categorized mapping of the charge carrier responses of (a). An outlier positioned far from all categories was colored in black (#0). (c) A microscopic image in the same area as (a). (d) The averaged responses for each category in (b) are shown. (red: category 1, blue: category 2, green: category 3)

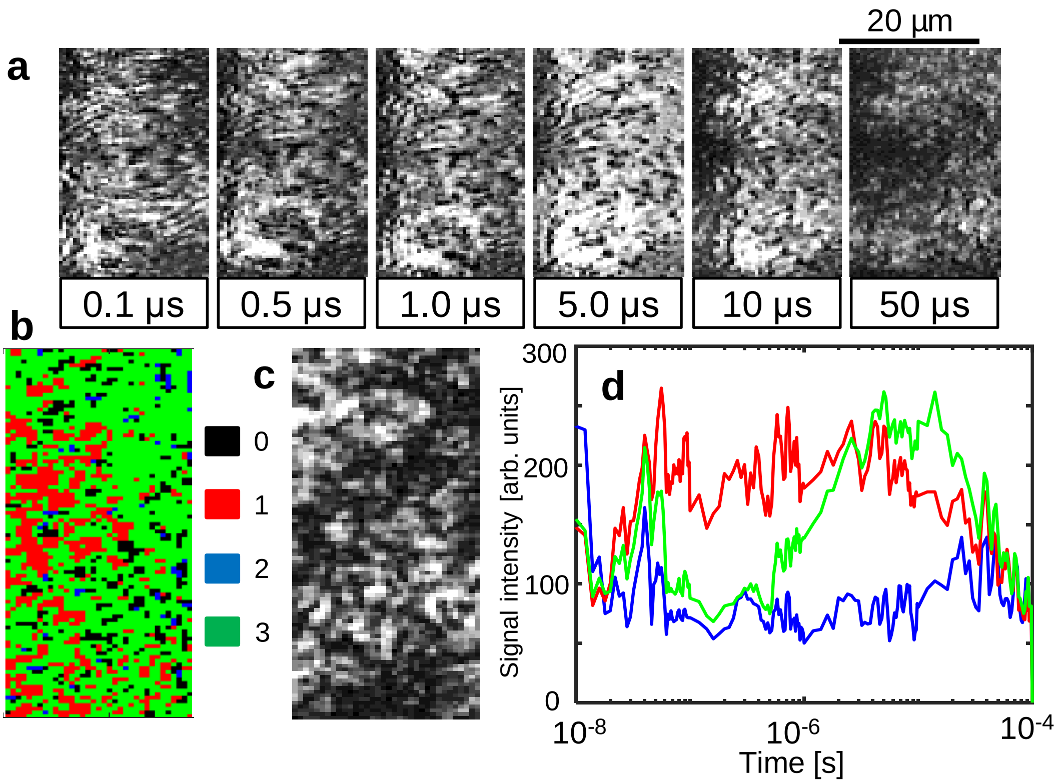

Supplementary Figure 7. **Image sequence and clustering analysis.** (a) An image sequence of the refractive index response for $SrTiO_3$:Rh/$BiVO_4$:Mo (STOR/BVOM) in acetonitrile (ACN) in a square region (18×50 µm) corresponding to No.2 in Fig.1(c) on the order from nanoseconds to microseconds. The scale bar corresponds to 20 µm. (b) The categorized mapping of the charge carrier responses of (a). An outlier positioned far from all categories was colored in black (#0). (c) A microscopic image at the same area as (a). (d) The averaged responses for each category in (b) are shown. (red: category 1, blue: category 2, green: category 3)

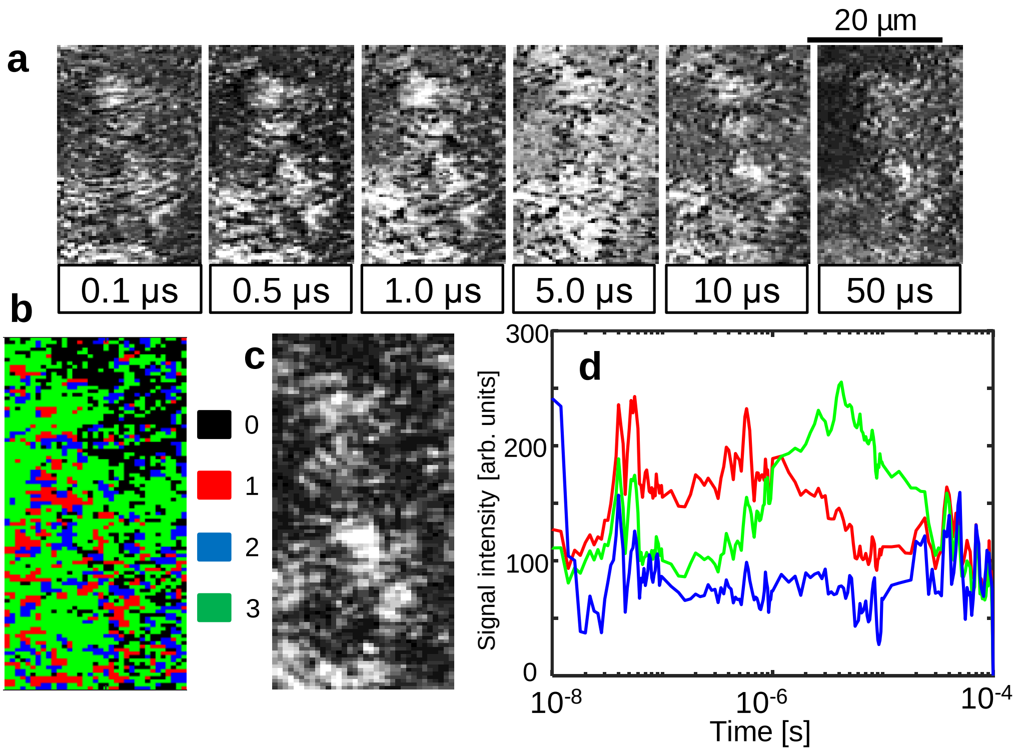

Supplementary Figure 8. **Image sequence and clustering analysis.** (a) An image sequence of the refractive index response for $SrTiO_3$:Rh/$BiVO_4$:Mo (STOR/BVOM) in acetonitrile (ACN) in a square region (18×50 µm) corresponding to region No.3 in Fig.1(c) on the order from nanoseconds to microseconds. The scale bar corresponds to 20 µm. (b) The categorized mapping of the charge carrier responses of (a). An outlier positioned far from all categories was colored in black (#0). (c) A microscopic image in the same area as (a). (d) The averaged responses for each category in (b) are shown. (red: category 1, blue: category 2, green: category 3)

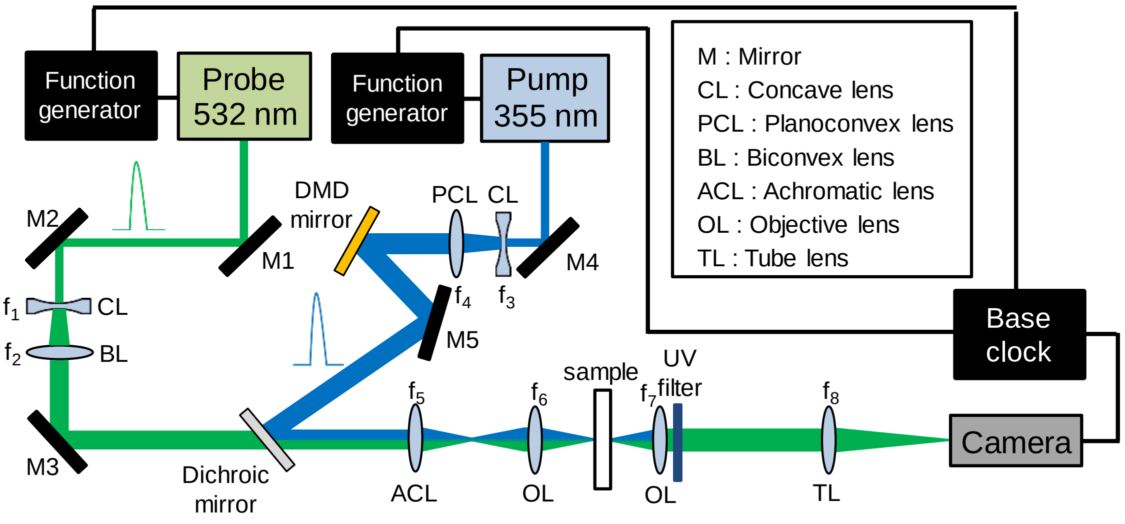

Supplementary Figure 9. **Schematic overview of the optical setup.** This schematic drawing represents the setup of the patterned-illumination time-resolved phase microscopy (PI-PM). For the pattern illumination, a digital micromirror device (DMD) (Light Crafter 4500, Texas Instruments) was used. The pump light was reflected at the DMD mirror to change the intensity pattern the same as the pattern on a computer. The image of the DMD mirror was relayed with a lens (f = 100 mm) and an objective lens (LUCPLFLN20x, Olympus) to irradiate the same pattern reduced in size (1/14) onto a sample. The pulsed illumination light was collimated with the pump light at the dichroic mirror and illuminated onto a sample. The transmitted light was imaged by an objective lens (LUCPLFLN20x, Olympus) and a tube lens (TTL180-A. Thorlabs). A CMOS camera (MV1-D1024E-160, Photon Focus) had a sensor area of 10.9x10.9mm (1024x1024 pixel) and the central region in the vertical direction (200x1024 pixel) was recorded to reduce the burden of the computer processing. The diameter of the irradiated area by the pump pulse was 0.5 mm. A sequence of images was stored in a computer by varying the time delay between the pump and probe pulse. The time resolution was limited only by the pulse width of the pump and probe lights, 3-5 ns. The pump light was the third harmonics of a Nd:YAG pulse laser (pulse width: 3-5 ns, wavelength: 355 nm) (GAIA, Rayture Systems). The probe light was the second harmonics of an Nd:YAG pulse laser (pulse width: 5 ns, wavelength: 532 nm) (GAIA, Rayture Systems). The timing of these pulses was controlled by two function generators (WF1968, NF) triggered by a base clock (DF1906, NF). Each function generator controlled both the timing of the flash lamp and the Q-switch with a time resolution of 100 ps. The pump light intensity was 2.23 mJ/pulse, and the probe light intensity was 0.02 mJ/pulse, respectively.

Preparation of photocatalyst sheets

Printed photocatalyst sheets were prepared as follows. Photocatalysts used, Rh-doped $SrTiO_3$ (Rh/(Rh+Ti) = 4 mol%) and Mo-doped $BiVO_4$ (Mo/(Mo+V) = 0.05 mol%), were prepared by the previous methods.[R1,R2] indium tin oxide (ITO) nanoparticles (ca. 20 nm) were purchased from Koshin Chemical. $SrTiO_3$:Rh, $BiVO_4$:Mo, and ITO were dispersed at a 2:2:1 mass ratio in the organic medium (α-terpineol : 2-(2-butoxyethoxy)ethanol : acrylic resion (SPB-TE1) = 4:10:2 (mass ratio). The mass ratio of the powder mixture to the organic medium was 1:19. Then the resulting paste was coated onto a glass substrate by screen-printing using a metal mask (60 μm thick) and finally calcined in air at 573 K for 30 min. The film thickness was approximately 1 μm.

(R1) Konta, R.; Ishii, T.; Kato, H.; Kudo, A. Photocatalytic Activities of Noble Metal Ion Doped SrTiO3 under Visible Light Irradiation. *J. Phys. Chem. B* **108**, 8992–8995, (2004).

(R2) Iwase, A.; Kudo, A. Photoelectrochemical Water Splitting Using Visible-Light-Responsive BiVO4 Fine Particles Prepared in an Aqueous Acetic Acid Solution. *J. Mater. Chem.*, **20**, 7536–7542, (2010).

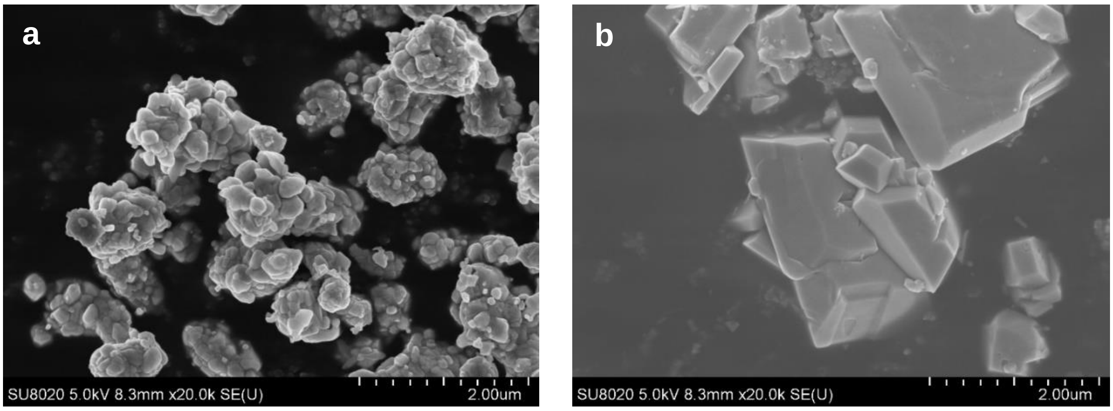

Supplementary Figure 10. **Microscopic images**. These images correspond to the scanning electron microscope (SEM) images of (a) $SrTiO_3$:Rh (STOR) and (b) $BiVO_4$:Mo (BVOM). The average diameters of particles were 300 nm and 2 μm, respectively.

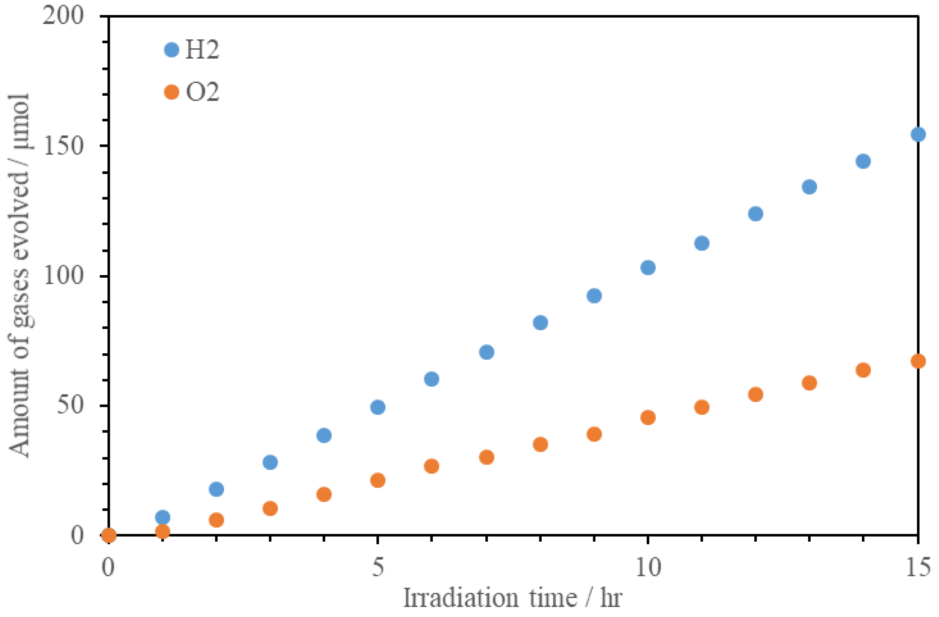

Supplementary Figure 11. **Time course of gas evolution.** The graph represents the time courses of amounts of gas evolution on a photocatalyst sheet under visible light irradiation. Sheet size, 2.5 × 2.5 cm; reactant solution, 40 mL of pure water at 288 K; reaction cell, top-irradiated separable cell; light source, 300 W Xe lamp with cut-off filters (λ > 410 nm) .

Phenomenological kinetic analyses for charge carrier dynamics in Z scheme materials

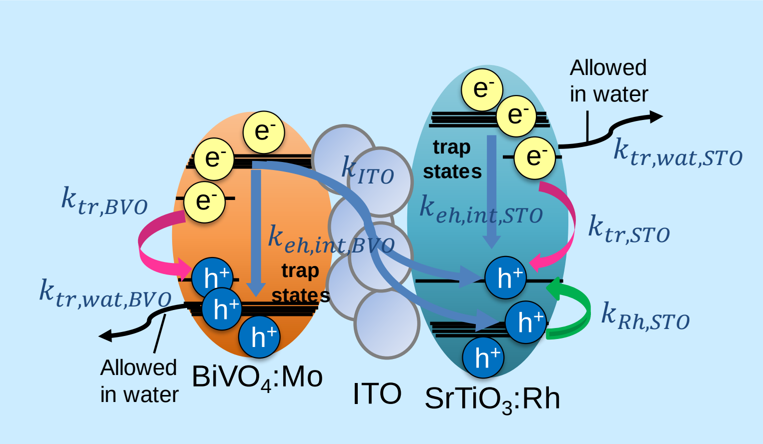

Supplementary Figure 12. **Schematic drawing for charge transfer analysis.** The scheme of the kinetic processes for the Z scheme system is shown, and the kinetic parameters are included in the figure.

The kinetics of the charge carriers are described. The whole processes considered are summarized in Supplementary Fig. 12. In BVOM,

$$\frac{dn_{eh,BVO}}{dt} = -k_{eh,int,BVO}n_{eh,BVO} \quad (1),$$

$$\frac{dn_{h,tr,BVO}}{dt} = k_{eh,int,BVO}n_{eh,BVO} - k_{tr,BVO}n_{e,BVO}n_{h,tr,BVO} - k_{tr,wat,BVO}n_{h,tr,BVO} \quad (2),$$

$$\frac{dn_{e,tr,BVO}}{dt} = k_{eh,int,BVO}n_{eh,BVO} - k_{tr,BVO}n_{e,tr,BVO}n_{h,tr,BVO} - k_{ITO}n_{e,tr,BVO}n_{h,tr,STO} \quad (3)$$

where $n_{eh,BVO}$, $n_{h,tr,BVO}$ and $n_{e,tr,BVO}$ correspond to the number of charge carriers for the bandgap states and the trapped hole and electron states, respectively. The kinetic parameters are described in Supplementary Fig. S12. Since the electrons and holes involved in the intrinsic recombination cannot be distinguished in our measurement, they were represented by the total number of charge carriers and approximated with a single kinetic parameter, although the actual recombination should be bimolecular-type recombination. In STOR,

$$\frac{dn_{eh,STO}}{dt} = -k_{eh,int,STO}n_{eh,STO} \quad (4),$$

$$\frac{dn_{h,tr,STO}}{dt} = k_{eh,int,STO}n_{eh,STO} - k_{Rh,STO}n_{h,tr,STO} - k_{ITO}n_{e,tr,BVO}n_{h,tr,STO} \quad (5),$$

$$\frac{dn_{e,tr,STO}}{dt} = k_{eh,int,STO}n_{eh,STO} - k_{tr,STO}n_{e,tr,STO}n_{Rh,STO} - k_{tr,wat,STO}n_{e,tr,STO} \quad (6),$$

$$\frac{dn_{Rh,STO}}{dt} = k_{Rh,STO} n_{h,tr,STO} - k_{tr,STO} n_{e,tr,STO} n_{Rh,STO} \quad (7),$$

where $n_{eh,STO}$, $n_{h,tr,STO}$, and $n_{e,tr,STO}$ corresponds to the number of charge carriers for the bandgap states and the trapped hole and electron states, respectively, and $n_{Rh,STO}$ is the number of charges trapped in the Rh states. The kinetic parameters are described in Supplementary Fig. S12.

In acetonitrile, the water-splitting reactions are blocked, and the second terms in Eq.(2) and (6) can be neglected. When BVOM and STOR are not combined to make a Z scheme material, or the charge transfer between two materials is inefficient without the ITO mediator, the charge compensation is hindered, and the second terms in Eq.(3) and (5) can be ignored.

In the simulation, the intrinsic recombination in BVOM and STOR was approximated with a single exponential decay. Actually, they cannot be described by a simple exponential function, and they consist of multiple exponential functions and are sometimes described by a stretched exponential function. The functional form can be recognized in Fig. 2(a) and (b) in water. However, they cannot be included in the analysis, and they were approximated as Eq.(1) and (4).

For the analysis, the rate for the intrinsic recombination for BVOM and STOR was estimated by using Fig. 2 **a** and **b**, and they were 3.5 and 1.0 (/s cm^{-3}), respectively. Then, Eq.(1)-(7) were solved simultaneously, and one of the simulated results is shown in Supplementary Fig.S13. The parameters were manually adjusted to match the observed responses peaked around 1 μs for water-splitting and 10 μs for the Rh state formation and decay. The charge carriers for water-splitting (electrons in STOR and holes in BVOM) were reproduced with a peaked around 1 μs, and the inactive state for water-splitting (Rh states) was reproduced with a peaked around 10 μs. The final refractive index response is made up of electron and hole responses of BVOM and STOR and the Rh state in STOR, and there are many parameters to adjust for reproducing the real signal, and we could not decide the appropriate combinations of parameters. However, we could confirm the effect of the charge compensation between BVOM and STOR via the charge mediator, ITO, by adjusting k_{ITO}. As shown in Supplementary Fig. S13. It clearly shows that the amplitude of the Rh states was much reduced by increasing the charge transfer rate. This simulation strongly supports that the ITO mediator suppressed the charge trapping at the Rh states.

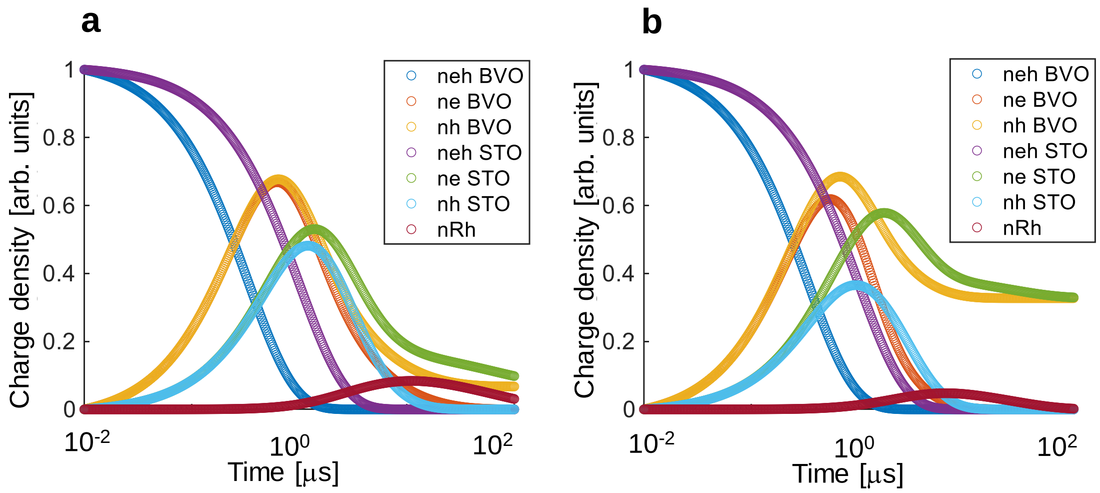

Supplementary Figure 13. **Simulation of charge carrier density change.** The simulated charge carrier densities for the Z scheme system are shown. The simulations were performed based on Eq.(1) to (7), and the used parameters were $k_{eh,int,BVO} = 3.5$, $k_{tr,BVO} = 1.0$, $k_{eh,int,STO} = 1.0$, $k_{tr,STO} = 1.0$, $k_{Rh,STO} = 0.1$, $k_{tr,wat,STO} = 0$, $k_{tr,wat,BVO} = 0$. (/µs cm^{-3}) Only the charge transfer rate by an ITO mediator, (a) $k_{ITO} = 0.1$, (b) $k_{ITO} = 1$.
